# Does orchidopexy for testicular torsion affect long‐term fertility parameters in males: A systematic review

**DOI:** 10.1002/bco2.70287

**Published:** 2026-10-18

**Authors:** Avula Aishwarya Rao, Muhammad Shariq Rahemtoola, Hassaan Aziz, Peter Grice, Mohamed Mubarak, Ian Pearce, Theodora Stasinou, Vaibhav Modgil

**Affiliations:** ^1^ Sheffield Medical School University of Sheffield Sheffield UK; ^2^ Manchester Andrology Research Collaborative (MARC) Manchester University NHS Foundation Trust Manchester UK; ^3^ Department of Urology Manchester Royal Infirmary Manchester UK

**Keywords:** fertility, orchidopexy, patency rates, pregnancy rates, sperm analysis, testicular torsion

## Abstract

**Objectives:**

The aim of this study is to investigate long‐term fertility outcomes following orchidopexy performed for testicular torsion.

**Patients and Methods:**

This systematic review adhered to the Preferred Reporting Items for Systematic Reviews and Meta‐Analyses (PRISMA) guidelines and was registered with the International Prospective Register of Systematic Reviews (PROSPERO: CRD420251152057). A literature search was conducted from June 2025 to October 2025 across Scopus, PubMed, Web of Science and the StarPlus Library (University of Sheffield). Studies reporting on male patients with a history of testicular torsion who underwent orchidopexy were included. Eligible study designs comprised case reports/series, cohort studies, case–control studies and randomised controlled trials. Studies involving cryptorchidism, orchidectomy or no intervention following exploration, as well as systematic reviews, meta‐analyses, editorials, book chapters, conference proceedings/posters, reviews and commentaries, were excluded.

**Results:**

Seventeen articles with 324 patients analysed fertility and hormonal outcomes following detorsion with orchidopexy. Primary outcomes were classified as fertility parameters and semen analysis. Reported pregnancy rates were high, ranging from 90.2% to 91.3% with reported time to pregnancy of 6.6 to 8.9 months. Six studies reported semen parameters with sperm count (11.2–235.4 × 10), motility (51.6–67%) and morphology (3.6–28%) generally within reported reference ranges, although substantial variability existed between studies. Secondary outcomes included hormonal analysis and anti‐sperm antibody prevalence. Five studies reported hormonal follicle‐stimulating hormone (FSH) and luteinising hormone (LH) levels, eight studies reported testosterone levels and two reported inhibin B levels. Anti‐sperm antibodies (ASA) were described in two studies, with positive ASA ranging from 20% to 27.2%. The reported follow‐up duration across nine studies was 0.12–11.8 years, although none reported fertility outcomes longitudinally.

**Conclusion:**

This review suggests that fertility potential is generally preserved following testicular detorsion with orchidopexy, with the majority of studies reporting favourable semen profiles and pregnancy outcomes. However, the small number of heterogeneous studies and limited long‐term data highlight the need for larger, standardised studies to better define the long‐term impact of testicular torsion on fertility.

## 1 INTRODUCTION

Testicular torsion (TT) is a urological emergency in which twisting of the testis along the axis of the spermatic cord leads to occlusion of venous outflow, reduced arterial inflow and subsequent testicular ischaemia.^1^, ^2^ TT can be classified as either extravaginal or intravaginal torsion, with the latter primarily affecting adolescent males and representing the majority of TT cases.^3^, ^4^ It is commonly associated with ‘bell clapper deformity’, an anatomical abnormality leading to abnormal fixation of the tunica vaginalis to the testis, thus increasing testicular mobility within the tunica vaginalis and increasing the possibility of TT.^3^, ^4^

Although TT has a relatively low incidence of approximately 1 in 4000 males, primarily affecting adolescent and young adult males, delayed treatment is associated with a marked risk of complications, including testicular atrophy, necrosis, morbidity and impaired fertility.^1^, ^2^ Prompt surgical intervention is essential to correct the torsion and restore blood flow. Management involves either orchidectomy, if the testis is nonviable, or manual detorsion with orchidopexy if salvageable. Although TT usually presents unilaterally, both the literature and the National Institute for Health and Care Excellence UK (NICE) guidelines recommend contralateral orchidopexy, as there is between 40% and 80% risk of torsion in the contralateral testis if fixation is not performed.^3^, ^4^, ^5^, ^6^ Studies also demonstrate a strong correlation between testicular salvage rate and the duration from symptom onset to surgical intervention: 90%–100% if treated within 6 h, 50% after 12 h and approximately 10% after 24 h.^3^

Although numerous studies have explored the surgical approaches to orchidopexy in cases of testicular torsion, particularly in relation to the prevention of retorsion, robust evidence regarding their long‐term impact on fertility remains scarce.^7^ The absence of randomised controlled trials limits the ability to establish causal relationships or draw definitive conclusions. Moreover, high‐quality, large‐scale research incorporating longitudinal follow‐up data on postoperative fertility outcomes is notably lacking. This study seeks to summarise and amalgamate existing literature on orchidopexy for testicular torsion, with a focus on postoperative outcomes associated with fertility. Given anticipated heterogeneity in the study design, patient age, timing of intervention, and outcome reporting, a narrative synthesis approach was adopted. We hypothesise that orchidopexy performed promptly after symptom onset is correlated with improved testicular viability, enhanced sperm parameters and better overall fertility outcomes.

## 2 MATERIALS AND METHODS

### 2.1 Protocol

This systematic review was conducted in accordance with the Preferred Reporting Items for Systematic Review and Meta‐Analysis (PRISMA) guidelines.^8^, ^9^ The study was registered with the International Prospective Register of Systematic Reviews (PROSPERO CRD420251152057).

The population, interventions, control and outcome (PICO) criteria are as follows:

P: Male patients who had testicular torsion.
I: Orchidopexy.
C: N/A.
O: Fertility parameters—semen analysis, sperm number, sperm function, sperm morphology, anti‐sperm antibodies, and patency and pregnancy rates.

### 2.2 Search strategy

The systematic search was conducted from 16 June 2025 to 27 October 2025 across three databases and one registry: Scopus, PubMed, Web of Science and StarPlus Library (University of Sheffield). The Booleans operands used the following combination of keywords: ‘testicular torsion’, ‘orchidopexy’, ‘surgery’, ‘fertility’, ‘infertility’, ‘sub‐fertility’, ‘anti‐sperm antibodies’ and ‘testicular fixation’ (Table 1). Two authors (HA and MSR) screened the eligible studies and performed the primary screening and data extraction. This was then reviewed independently by a third author (AAR) to address conflict and verify inclusion of studies in the paper. Articles were retrieved from journals and uploaded to Covidence. After initial title and abstract screening, all relevant articles that met the inclusion criteria were retrieved.

**TABLE 1:** Demographics of included studies.

Author	Year	Study design	Sample size	Number of orchiopexy	Mean age of torsion in years
Almekaty et al.^10^	2023	Prospective cohort	62	20	‐
Al‐Nasiri et al.^11^	2010	Cross‐sectional	17	5	13.0
Anderson et al.^12^	1986	Prospective cohort	56	43	17.0
Anderson et al.^13^	1992	Retrospective cohort	16	9	18.6
Arap et al.^14^	2007	Cross‐sectional	24	9	15.0
Gielchinsky et al.^15^	2016	Retrospective observational	63	41	20.3
Hansen et al.^16^	2023	Cross‐sectional	57	52	13.4
Mäkelä et al.^17^	2020	Cross‐sectional	35	16	13.0
Newman et al.^18^	2024	Retrospective case–control	30	23	16.6
Rasmussen^19^	1996	Case Series	4	4	9.5
Romeo et al.^20^	2010	Prospective cohort	20	12	‐
Thomas et al.^21^	1983	Prospective clinical study	80	22	21.0
Törzsök et al.^22^	2022	Prospective cohort	49	20	18.0
Tryfonas et al.^23^	1994	Retrospective cohort	25	18	6.6
Woodruff et al.^24^	2010	Case report	1	1	13.0
Zhang et al.^25^	2020	Retrospective cohort	72	23	‐
Zilberman et al.^26^	2006	Retrospective case series	11	6	0.6

*Note*: ‐ means not reported.

Data were extracted into an Excel spreadsheet, and included studies were analysed for patient demographics and outcomes. The study demographics analysed included study design, date of publication, sample size and number of orchidopexies. Because of substantial heterogeneity in study populations, outcome definitions, semen analysis methodology, hormonal reporting and follow‐up duration, a formal meta‐analysis was not performed. The findings were instead synthesised narratively.

### 2.3 Outcomes

The outcomes were divided into two groups—primary outcomes and secondary outcomes. Primary outcomes consisted of fertility parameters and seminal analysis, whereas secondary outcomes consisted of hormonal levels, anti‐sperm antibodies (ASA) and follow‐up.

The reported time‐to‐pregnancy outcomes were converted into months for consistency. Semen parameters were extracted as reported by individual studies.

- Fertility parameters: Paternity rate (*n* [%]), pregnancy rate (*n* [%]) and time to pregnancy (month; mean ± standard deviation).
- Seminal analysis: Sperm count (×10^6^), sperm motility (%) and sperm morphology (%)
- Hormonal analysis: FSH levels (IU/L), LH levels (IU/L), inhibin B levels (pg/mL) and testosterone levels (nmol/L)
- ASA (%)
- Follow‐up.

The risk of bias in non‐randomised studies of interventions (ROBINS‐I) tool was used to evaluate the risk of bias which is represented visually using the ROBINS tool in Figure 1.^27^

**FIGURE 1:**
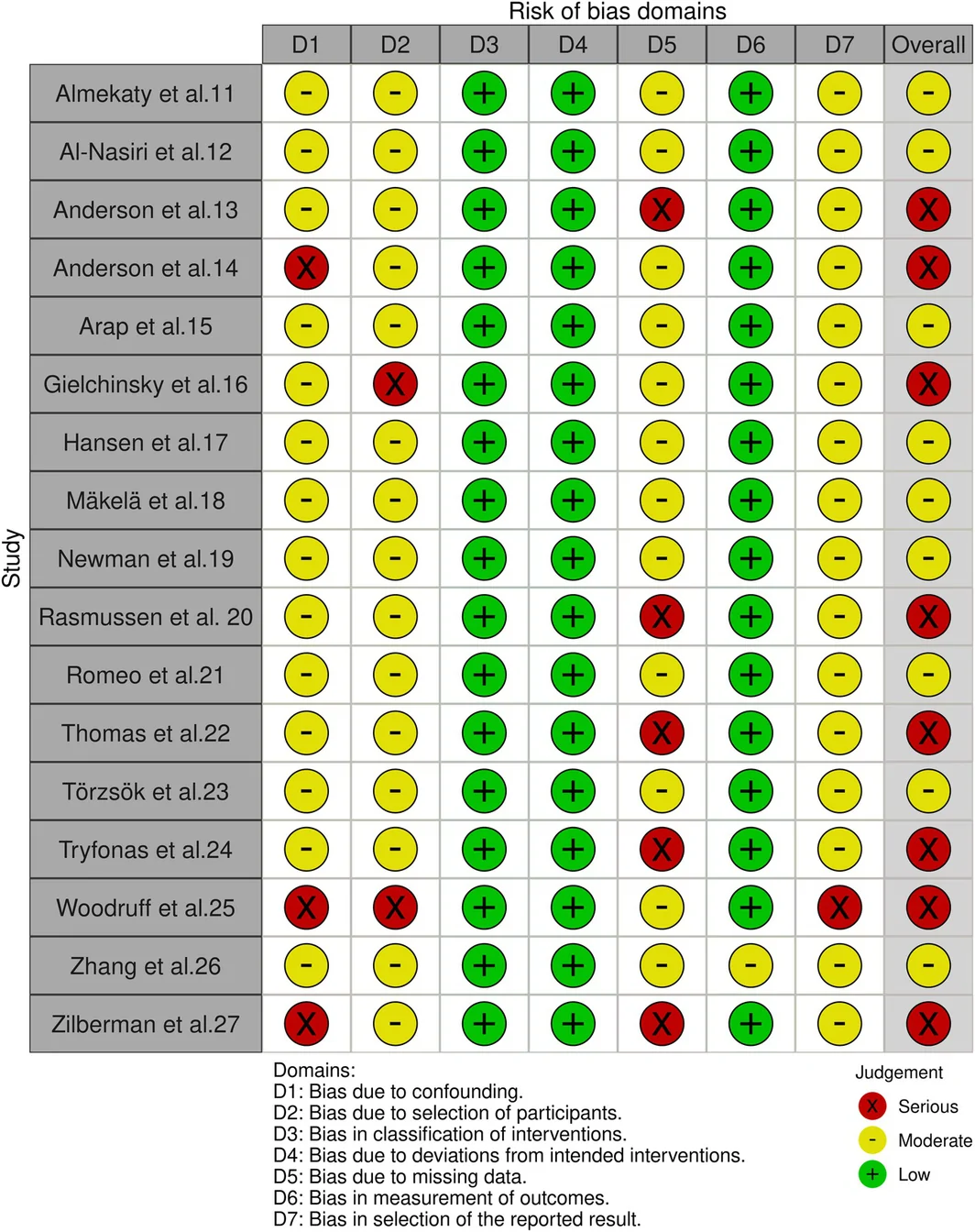
ROBINS‐I risk of bias assessment for the included nonrandomised studies. ROBINS‐I, risk of bias in non‐randomised studies of interventions.

### 2.4 Inclusion and exclusion criteria

Articles included in this systematic review were published in English in peer‐reviewed journals between 1986 and 2024. Inclusion criteria comprised male patients with a history of testicular torsion who underwent orchidopexy. Eligible study designs included case reports, case series, cohort studies, case–control studies and randomised controlled trials.

Exclusion criteria included males diagnosed with cryptorchidism and those who underwent orchidectomy or received no intervention upon exploration. The following publication types were also excluded: meta‐analyses, systematic reviews, editorials, book chapters, posters, conference proceedings, reviews and commentaries.

## 3 RESULTS

### 3.1 Study selection

The initial search identified 106 studies. Using Covidence, 21 duplicates were removed, and a further 66 studies were excluded following title, abstract and full‐text screening. In total, 17 studies met the inclusion criteria, and data from these were extracted into an Excel spreadsheet. The study selection process is illustrated in Figure 2.

**FIGURE 2:**
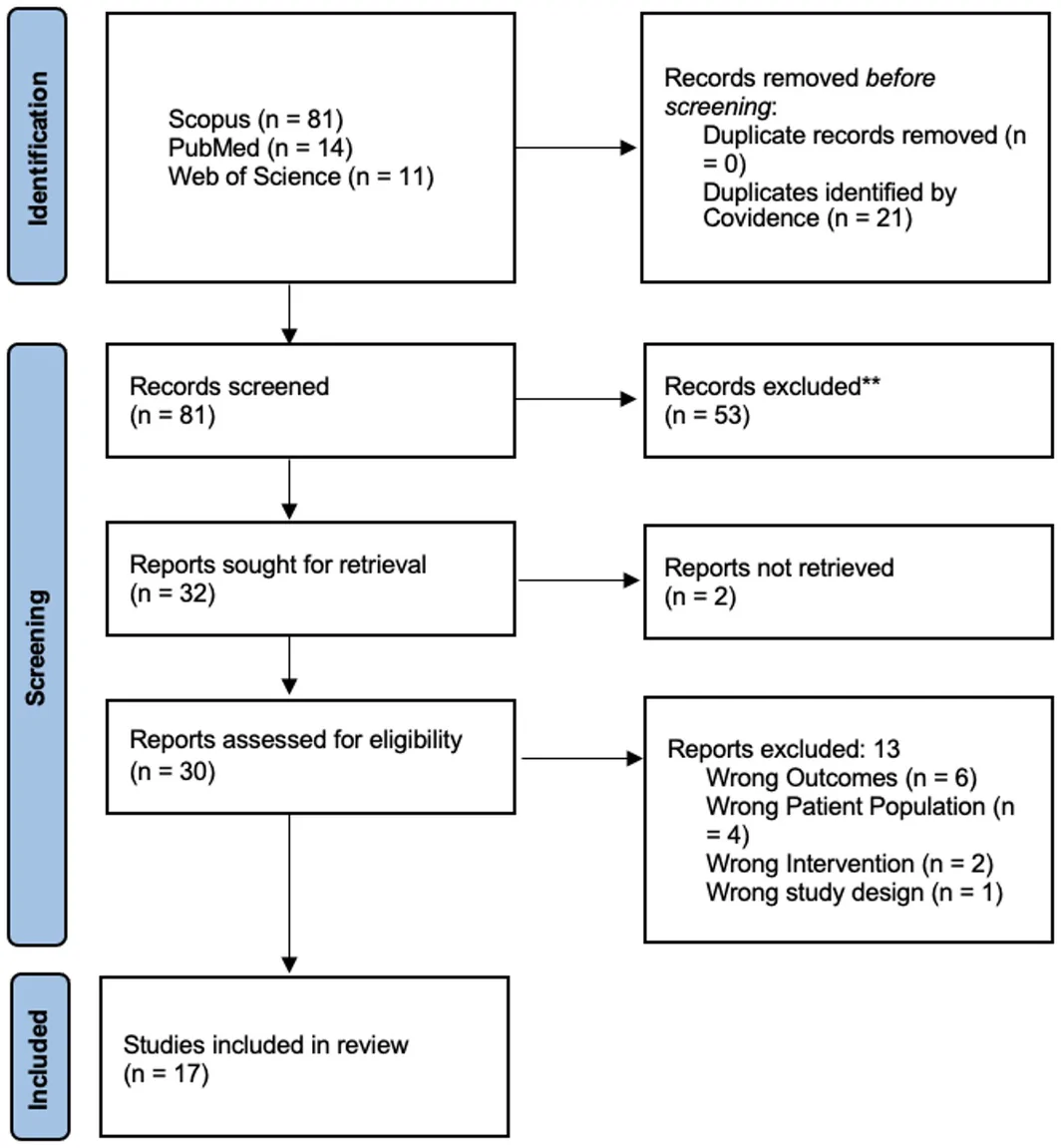
PRISMA flow diagram illustrating the study selection process. PRISMA, Preferred Reporting Items for Systematic Review and Meta‐Analysis.

These 17 studies consisted of six prospective cohort studies, four cross‐sectional studies, two retrospective cohort studies, two case series, one case report, one retrospective case–control study and one retrospective observational study. The included studies were published between 1983 and 2024 in peer‐reviewed journals (Table 2).

**TABLE 2:** Fertility parameters.

Author	Year	Paternity rate, *n* (%)	Pregnancy rate, *n* (%)	Mean time to pregnancy in years
Gielchinsky et al.^15^	2016	‐	37 (90.2)	0.55
Mäkelä et al.^17^	2020	13 (81)	‐	‐
Zhang et al.^25^	2020	‐	21 (91.3)	0.75

*Note*: ‐ means not reported.

### 3.2 Demographical data

Seventeen studies comprising 622 patients analysed fertility parameters for 324 detorsions with orchidopexy. Patients who underwent orchidectomy with contralateral orchidopexy were excluded. Fifteen studies reported patient age, with a mean of 14.0 years and a range of 2 weeks to 33 years (Table 2).

Ten included studies reported contralateral orchidopexy in all patients undergoing surgery for testicular torsion.^10^, ^11^, ^12^, ^13^, ^14^, ^15^, ^18^, ^21^, ^22^, ^23^ Rasmussen et al. described patients who had previously undergone orchidopexy for unilateral cryptorchidism and subsequently developed torsion of the previously unaffected, normally descended contralateral testis, which was treated with orchidopexy.^19^ This study was included because it specifically reported orchidopexy performed for testicular torsion of a previously unaffected testis, thereby meeting the review's inclusion criteria.

### 3.3 Ischaemia duration and time to intervention

Ten studies reported time to detorsion for patients undergoing orchidopexy. Median symptom‐onset‐to‐surgery times were 7 h in Al‐Nasiri et al. and Arap et al. (IQR 3–21 h in the latter), and 6 h (IQR 4–11 h) in Mäkelä et al.^11^, ^14^, ^17^ Mean times were 13.0 ± 15.8 h in Anderson et al. and 3.0 ± 1.7 h in Törzsök et al.^13^, ^22^ Thomas et al. reported a range of 3–24 h, whereas Zhang et al. reported that 33 surgically preserved testes underwent orchidopexy within 6 h of torsion onset.^21^, ^25^ Anderson and Williamson reported individual operative times of ≤2, 3.5 and 5 h.^12^ Rasmussen reported case‐specific symptom durations of 2–12 h; one patient underwent manual detorsion after 4 h and orchidopexy the following day.^19^ Woodruff et al. described a solitary‐testis case in which surgery was performed within 0.5 h of notification following presentation 9 h after symptom onset.^24^

Quantitative pooling of operative‐timing data was not undertaken because of heterogeneity in timing definitions, summary measures and study populations. Orchidopexy‐specific median symptom‐to‐surgery times were nevertheless consistent across the three studies reporting this measure, ranging from 6 to 7 h. The remaining studies were synthesised descriptively because they reported means, ranges, thresholds or individual‐case data.

Zhang et al. was the only study to stratify outcomes by age at torsion (<14, 14–18 and >18 years), although operative timing was not reported separately for these groups.^25^ Almekaty et al. and Anderson et al. included exclusively post‐pubertal patients.^10^, ^13^ The remaining studies did not report outcomes stratified by age or pubertal status, precluding further quantitative subgroup analysis.

### 3.4 Primary outcomes

#### 3.4.1 Fertility parameters

Fertility parameters analysed included paternity rate (%), pregnancy rate (%) and time to pregnancy (years). Three studies reported fertility outcomes. Zhang et al. reported a pregnancy rate of 91.3% (21/23) with a time to pregnancy of 8.9 ± 1.3 months. ^[25^ ^]^ Gielchinsky et al. observed a similarly high pregnancy rate of 90.2% with a time to pregnancy of 6.6 ± 5.5 months. ^[15^ ^]^ Mäkelä et al. reported a paternity rate of 81% but did not provide data on pregnancy rate or time to pregnancy.^17^ The reported time to pregnancy ranged from 6.6 to 8.9 months across the two studies reporting this outcome (Table 3).

**TABLE 3:** Seminal analysis.

Author	Year	Sperm count (×10^6^)	Sperm motility (%)	Sperm morphology (%)
Al‐Nasiri et al.^11^	2010	33.8	58.2	26.0
Arap et al.^14^	2007	47.0	54.0	7.0
Hansen et al.^16^	2023	147.0	59.0	6.5
Newman et al.^18^	2024	108.6	51.6	3.6
Törzsök et al.^22^	2022	235.4	62.0	12.0
Woodruff et al.^24^	2010	11.2	67.0	28.0

#### 3.4.2 Semen analysis

Seminal parameters analysed included sperm count (×10^6^), sperm motility (%), sperm morphology (%) and ASA (%). Six studies reported on semen parameters following detorsion and orchidopexy. The reported sperm count values varied considerably across studies, ranging from 11.2 to 235.4 × 10^6^. The same six studies also reported sperm motility ranging from 51.6% to 67%, whereas sperm morphology ranged from 3.6% to 28% (Table 3).

Variability in semen analysis methodology, reporting units and reference standards limited direct comparison between studies.

### 3.5 Secondary outcomes

#### 3.5.1 Hormonal summary

Hormonal parameters analysed included follicle‐stimulating hormone (FSH; IU/L), luteinising hormone (LH; IU/L), inhibin B (pg/mL) and testosterone (nmol/L). Five studies reported FSH and LH levels, whereas eight studies reported on testosterone levels. The reported hormonal values varied across studies (table) but were generally within expected laboratory reference ranges. The reported FSH was in the range of 2.7–6.3 IU/L and LH levels in the range of 2.2–6.6 IU/L. Anderson et al. (1992) was excluded from FSH and LH analyses because of reporting data in nonstandard units.^13^ With regards to the testosterone level, it was noted to be a range of 14.5–22.4 nmol/L. Hansen et al. and Romeo et al. reported inhibin B levels with a mean of 100.7 ± 70.3 pg/mL and a range of 30.4–171.0 pg/mL.^16^, ^20^ Interpretation was limited by inconsistent age‐adjusted reporting and variability in hormonal assessment methods across the different studies.

#### 3.5.2 Anti‐sperm antibodies

Two studies, Al‐Nasiri et al. and Arap et al., reported anti‐sperm antibody positivity rates (%) with a range of 20%–27.2%^11^, ^14^ (Table 4).

**TABLE 4:** Hormonal analysis, ASA and follow‐up.

Author	Year	FSH levels (IU/L)	LH levels (IU/L)	Inhibin B levels (pg/mL)	Testosterone levels (nmol/L)	Anti‐sperm antibodies (%)	Follow‐up duration in years
Almekaty et al. ^10^	2023	6.3	6.6	‐	18.9	‐	0.12
Al‐Nasiri et al. ^11^	2010	5.0	2.9	‐	22.4	27.2	‐
Anderson et al. ^12^	1986	‐	‐	‐	‐	‐	0.5
Anderson et al. ^13^	1992	216.2 ± 93.63 ng/mL ^a^	24.7 ± 16.17 ng/mL ^a^	‐	19.3	‐	2.9
Arap et al. ^14^	2007	5.6	2.6	‐	22.2	20.0	10.0
Hansen et al. ^16^	2023	3.1	3.6	171.0	19.5	‐	‐
Romeo et al. ^20^	2010	2.9	2.2	30.4	14.5	‐	5.0
Thomas et al. ^21^	1983	‐	‐	‐	‐	‐	3.5
Törzsök et al. ^22^	2022	2.7	4.2	‐	15.5	‐	8.9
Tryfonas et al. ^23^	1994	‐	‐	‐	‐	‐	11.8
Woodruff et al. ^24^	2010	‐	‐	‐	14.8	‐	0.2

*Note*: ‐ means not reported.

^a^ Excluded as not standardised.

#### 3.5.3 Length of follow‐up

Length of follow‐up was reported by nine articles. The reported follow‐up duration ranged from 0.12 to 11.8 years. None of the studies reporting on length of follow‐up reported on fertility parameters (Table 4).

## 4 DISCUSSION

This systematic review demonstrates that fertility appears to be largely preserved after detorsion and orchidopexy. Across the three studies reporting fertility outcomes, pregnancy rates were high: 90.2%–91.3% in two cohorts and an 81% paternity rate in one cohort, with reported time to pregnancy ranging from 6.6 to 8.9 months (Table 3). In comparison, the cumulative conception rates in the general population as reported by Taylor (2003) was found to be around 75% after 6 months, 90% after a year and 95% at 2 years.^28^ Moreover, the time to pregnancy results of the German prospective study by Gnoth et al. showed a cumulative probability of conception of 81% after 6 months and 92% at 12 months in the general population.^29^ The figures from the general population indicate that time to pregnancy and the rate of conception were comparable to those reported across all three studies, that is, conception within the 12‐month window.^28^, ^29^

Seminal parameters, including sperm count, motility and morphology, across the studies were generally within or above normal ranges defined by the World Health Organisation.^30^ In fact, the reported sperm count across the five out of six studies reporting it was in a range of 33.8–235.4 × 10^6^ (Table 3), which is higher than the WHO (2021) lower reference limit of 16 × 10^6^ of ejaculate.^30^ However, direct comparison is limited by methodological heterogeneity. Where studies reported sperm concentration rather than total sperm count, values were extracted as reported and direct comparison between studies should therefore be interpreted cautiously.

Available hormonal data (FSH, LH and inhibin levels) did not consistently demonstrate overt endocrine dysfunction following orchidopexy; however, interpretation remains limited by heterogeneous reporting and lack of age‐matched controls (Table 4). ASA were present in 20%–27.2% of patients in the two studies reporting them (Table 4). This raises the important debate on whether ASA has an impact on fertility, what is the impact and what is the prevalence of ASA in the general fertile and infertile population. In a study on fertility by Garcia et al., it was noted that 18.18% of infertile men were positive for ASA, whereas no positivity was found in fertile men.^31^ Moreover, a study by Barbonetti et al. (2019) with a sample size of 10 025 showed a 3.4% prevalence of ASA in infertile men, and Lotti et al. (2018) with a sample size of 181 showed a 6.6% prevalence of ASA in infertile men.^32^, ^33^ A review of literature on the impact of anti‐sperm antibodies on human male reproductive function showed a mean ASA prevalence among the infertile male population between 2.6% and 6.6%.^34^ However, it is important to note that Lotti et al. (2018) study did also report a 0.9% prevalence of ASA among fertile men (*n* = 109).^30^, ^33^ This percentage is in keeping with similar studies that have been performed previously, with a significant prevalence of ASA in infertile men (5%–15%) and only 1%–2% of fertile men.^34^

The impact of ASA on male fertility can be in several ways. It is mentioned in the literature that ASA can lead to decreased sperm motility, promote sperm agglutination and impair sperm ability to penetrate the cervical mucus. ASA can also disrupt key functional processes, such as sperm capacitation, the acrosome reaction, binding to the egg and even the earliest phases of embryo development.^34^ Other sperm parameters, such as sperm viability, morphology and sperm count, can also be at risk of change in ASA positive male patients.^34^ The effects of ASA on motility tend to present immediately and can be quantified, and motility is therefore one of the most accepted altered parameters to the negative effects of ASA presence.^34^

It is, therefore, important to note that the prevalence of ASA in this review appears to be higher than the general male fertile population despite preservation in fertility and endocrine function in patients undergoing orchidopexy after detorsion in this study.

Moreover, the link between ASA and impaired DNA integrity remains unclear, although the link between sperm DNA damage and male infertility is well recognised.^35^, ^36^ Some studies have reported no association between ASA and impaired DNA integrity, whereas others have found a higher DNA fragmentation and an increase in sperm reactive oxygen species generation in men with ASA.^37^, ^38^ Overall, ASA can influence fertility by lowering semen quality, disrupting key steps in fertilisation, or through a combination of mechanisms, yet they do not cause infertility in all affected men.^34^, ^35^, ^36^, ^37^, ^38^ As research begins to reveal the specific sperm proteins targeted by ASA, deeper molecular understanding may improve the diagnosis of ASA‐related infertility and help guide more personalised treatment approaches.^34^

Moreover, studies comparing orchidectomy versus orchidopexy in unilateral TT have revealed that orchidopexy has generally been associated with more favourable fertility outcomes (pregnancy rate, median time to pregnancy).^25^ These studies suggested that viable surgical fixation of the testes yielded higher pregnancy rates and shorter time to pregnancy when compared to orchidectomy.^25^

## 5 LIMITATIONS

A major limitation of this review was the substantial clinical and methodological heterogeneity between included studies, including variation in patient age, pubertal status, ischaemia duration, semen analysis methodology, hormonal assessment and follow‐up duration. This precluded formal quantitative meta‐analysis and limited direct comparison between studies. The ROBINS‐I assessment highlights important sources of bias across the included nonrandomised studies evaluating outcomes following orchidopexy after testicular torsion. Studies, such as Anderson et al., Geilchinsky et al., Woodruff et al. and Zilberman et al., demonstrated serious overall risk of bias, largely attributable to uncontrolled confounding and substantial missing data.^13^, ^15^, ^24^, ^26^ Consequently, their reported postoperative outcomes, whether relating to fertility outcomes or seminal analysis, should be interpreted with caution, as these methodological limitations may impede the generalisability of their findings to the wider population undergoing orchidopexy.

Several other studies, including those by Anderson et al., Rasmussen et al., Thomas et al. and Tryfonas et al., exhibited serious concerns within individual domains, particularly participant selection and incomplete outcome reporting.^12^, ^19^, ^21^, ^23^ Although informative, such weaknesses reduce the internal validity of their conclusions.

In contrast, majority of the studies, such as Almekaty et al., Al‐Nasiri et al., Arap et al., Hansen et al., Mäkelä et al., Newman et al., Romeo et al., Törzsök et al. and Zhang et al., demonstrated predominantly moderate risk of bias, with consistently low risk in intervention classification and delivery.^10^, ^11^, ^14^, ^16^, ^17^, ^18^, ^20^, ^22^, ^25^ Their findings on postoperative outcomes are thus comparatively more robust and more likely to reflect the broader clinical population, despite some remaining vulnerability to residual confounding and selective reporting.

Another significant limitation of this review is that only 17 studies (324 patients) were identified, comprising differing designs (prospective, retrospective and case series), with considerable heterogeneity in outcome measurements, limiting the feasibility of meta‐analysis. Only three studies explicitly reported on conception, and none of the nine studies that reported follow‐up duration included fertility outcomes. This is of significance given that the fertility outcome assessment is based on a positive pregnancy, and therefore, most studies did not report the only clinically significant outcome. This is a major limitation to deriving meaningful conclusions from the evidence.

Additionally, seminal parameters were reported inconsistently, with differences in assessment timing, abstinence requirements, laboratory methods and outcome definitions limiting comparisons between studies. Analyses were also frequently restricted to participants who attended follow‐up and provided a semen sample. Thus, selection bias cannot be excluded and should be considered when assessing the generalisability of these findings.

The inclusion of case reports and small case series reduced the overall strength of evidence but was considered necessary because of the limited available literature on long‐term fertility outcomes following testicular torsion salvage.

Furthermore, when reporting on conception, partner‐related factors, such as age and fertility status, were also absent. This is of importance as the opposite gender infertility has an impact on conception rates and should be therefore taken into account when reporting the clinically significant outcome. Likewise, the studies have not compared the outcomes of orchidopexy on the contralateral (non‐twisted) testes versus the affected testes.

## 6 CONCLUSION

From the available data on conception and sperm parameters from the studies, it suggests that fertility may be preserved following successful detorsion and orchidopexy in selected patients. However, conclusions remain limited by small sample sizes, heterogeneous methodologies, inconsistent outcome reporting and limited long‐term fertility data. It was also noted that the ASA prevalence was higher in the patients undergoing detorsion and orchidopexy when compared to the general population. However, large‐scale studies with longer follow‐up and controlled variables are needed evaluate the impact of ASA on fertility and to determine the prevalence of ASA in the orchidopexy population after a testicular torsion, as only two studies reported on this.

## AUTHOR CONTRIBUTIONS

Hassaan Aziz and Muhammad Shariq Rahemtoola performed the initial literature search that was reviewed by Avula Aishwarya Rao for conflict and inclusion. Avula Aishwarya Rao and Muhammad Shariq Rahemtoola subsequently drafted the original version of the manuscript. Peter Grice, Mohamed Mubarak, Vaibhav Modgil, Ian Pearce and Theodora Stasinou reviewed and revised the manuscript prior to submission. Order of authorship was based on overall contribution to the project. All authors read and approved the final manuscript. All authors made significant contributions to the work and can claim authorship according to the conditions posed.

## CONFLICT OF INTEREST STATEMENT

The authors declare no conflicts of interest.

## Data Availability

The data that support the findings of this study are available from the corresponding author upon reasonable request.
